# The inter-familiar issues of Greek parents facing childhood cancer

**DOI:** 10.1007/s00431-023-05266-9

**Published:** 2023-10-21

**Authors:** Kleanthis Nizamis, Vassilios Kalliakmanis, Nikos Koutsoupias, Sophia Polychronopoulou, Margarita Baka, Evgenia Papakonstantinou, Emmanouil Hatzipantelis

**Affiliations:** 1 School of Theology, Aristotle University of Thessaloniki, Thessaloniki, Greece; 2 Department of International and European Studies, University of Macedonia, Thessaloniki, Greece; 3 Department of Pediatric Hematology-Oncology, Aghia Sophia Children’s Hospital, Athens, Greece; 4 Department of Pediatric Oncology, General Children’s Hospital of Athens Panagiotis & Aglaia Kyriakou, Athens, Greece; 5 Pediatric Oncology Department, Hippokration General Hospital of Thessaloniki, Thessaloniki, Greece; 6 Children’s & Adolescent’s Hematology-Oncology Unit of 2nd Paediatric Clinic, School of Medicine, Aristotle University of Thessaloniki, Thessaloniki, Greece

**Keywords:** Childhood cancer, Parents, Physical problems, Psychosocial aspects, Intra-family issues, Parents’ behavior, Quality of life

## Abstract

Cancer as a whole, but especially childhood cancer, creates a number of psychological, social, and family problems as well as practical and financial issues, which every parent is called upon to solve. This study focuses on childhood cancer and aims at a thorough analysis of the physical/organic, psychological, and social problems associated with the parents and relatives of a child with cancer. The special element in pediatric neoplasms is not only the vulnerable population target group, but also the set of secondary effects it has on the environment of the sick child. The research was conducted on a sample of 133 families of children with cancer, and the results were displayed after statistical processing and data analysis with R statistical software. The results of the study confirm with statistically significant data the effect of childhood cancer on the physical, mental, and social health and behavior of the parent. Thus, 53.8% of the respondents stated 5 and above on the 7-point Likert scale for fatigue issues, 55.6% for sleep disorders, 78.1% for stress, and 82.7% for fear. The key findings are characterized by high specificity as it is a unique study that reveals particular aspects of the Greek parent’s behavior, mind, and body during the period of their child’s illness.

*Conclusion*: The effects of childhood illnesses, particularly when they are severe, such as neoplasms, present a looming threat, ushering in a multitude of adverse alterations in the daily lives of the affected child's family.

**Table:** 

**What is Known – What is New:** *• We know the effects that a childhood illness brings not only to the sick child, but also to the entire family circle. The new element in the present research is that these data reflect the situation in Greece, for which the research data in this area is quite limited. Our research is one of the few studies that demonstrate with statistical data the change in the psychosomatic health of the parent who has a child with cancer.*

**What is Known – What is New:**

*• We know the effects that a childhood illness brings not only to the sick child, but also to the entire family circle. The new element in the present research is that these data reflect the situation in Greece, for which the research data in this area is quite limited. Our research is one of the few studies that demonstrate with statistical data the change in the psychosomatic health of the parent who has a child with cancer.*

## Introduction

Cancer is caused by the rapid proliferation of pathogenic cells, which divide uncontrollably and form tumors that work harmfully for the body. It is widely accepted that the evaluation of suspicious symptoms and findings is the basis for early diagnosis and subsequent successful treatment. The scientific development and medical expertise of recent years have made clear that cancer is no longer automatically translated as death, especially childhood cancer, in which the rates of treatment in developed countries are extremely high [1, 2].

About 280,000 children and adolescents aged 0 to 19 are being diagnosed with cancer each year, with the most common types of cancer at this age being leukemias, brain cancer, lymphomas, and solid tumors, such as neuroblastoma and Wilms tumors [3, 4]. In Greece, around more than 300 children are diagnosed with cancer every year, with the most common form being leukemia and the next two prevalent cancer diagnoses among Greek children being brain cancer and Hodgkin (HL) and non-Hodgkin (NHL) lymphomas [5, 6]. It is worth saying that the treatment rates between developed and non-developed countries differ significantly, with this difference reaching almost double the figure. Specifically, it is estimated that in high-income countries, where health services are generally accessible to the citizenry, more than 80% of children with cancer are treated, while in low- and middle-income countries (LMIC), the corresponding treatment rate is only 15–45% [7]. Despite the development of medical science and the provision of high health services, economic and social inequalities affect and shape the levels of child mortality due to cancer.

The special element in childhood neoplasms is not only the vulnerable population group it targets, but the set of secondary effects it brings to the sick child’s environment. Cancer, especially childhood cancer, creates a multitude of psychological, social, and intra-family problems as well as corresponding practical and financial issues, which each parent is called upon to resolve [8]. The scientific community agrees that most chronic illnesses, regardless of which person are associated with, have similar effects on family members, including psychological and emotional disturbances, disruption of leisure activities, impact on interpersonal relationships, and of course financial problems [8].

In this study, the answers that were collected from 133 families of children with cancer in Greece present a thorough analysis of the physical, psychological, social, and practical problems associated with the parents and the effect of childhood cancer on their behavior.

## Materials and methods

In Greece, we have about 300 cases of childhood cancer every year. As it was mentioned, the research was conducted on a sample of 133 families of children with cancer, and the results were displayed after statistical processing and data analysis with R statistical software. It is therefore easily understood that the research covered more than 1/3 of the total annual population of childhood cancer that exists in the country.

Four pediatric oncology departments of Greek hospitals took part in this study. More specifically, we studied 64 cases from the Pediatric Oncology Department of the “Hippocration” General Hospital of Thessaloniki, 26 from the Children’s & Adolescent’s Hematology-Oncology Unit of 2nd Paediatric Clinic of A.U.Th. in the AHEPA General University Hospital of Thessaloniki, 25 from the Oncology Department of the “Panagiotis & Aglaia Kyriakou” Children’s Hospital, and 18 from the Department of Pediatric Hematology-Oncology of “Agia Sophia” Children’s Hospital.

The aim of the empirical research was to gather questionnaires within 1 full calendar year, in order to collect a sample based on the total cases of childhood cancer that we have in our country. Despite the difficulty of the COVID pandemic, we accomplished collecting the data in a frame of 12 months of research, so the result was characterized by high representativeness. Participation in the research was carried out through the completion of a closed questionnaire, which was processed statistically in order to provide findings and results. For the compilation of the questionnaire, researches of other universities and research centers were taken into account on issues of management of childhood illness and specifically of neoplasia by parents and relatives.

In the main part of the questionnaire, a series of statements regarding the period of the child’s health issue is listed, in which each participant notes the extent to which each statement applies to him, expressing his agreement on a 7-point Likert scale. In addition, there are some questions in the form of multiple choices. The questionnaire consists of questions focusing on psychosocial and physical issues as well as practical and everyday issues. For example, there are questions like “after the onset of my child’s illness I faced fatigue or sleep disorders” or “during my child’s illness period I felt desperation or I felt significant fluctuations in my mood.” Statistical processing of the data was carried out by multivariate analysis, using methods of multiple correspondence analysis in a 0–1 table and automatic hierarchical classification. These methods were chosen as they allow the phenomenon to be examined as a whole, without assumptions and models [9, 10]. Τhe primary outcomes showed a significant development of both physical and psychological problems, while parallel improvement of the couple’s relationship during the period of childhood illness.

## Results

From the study, statistically significant results were found, mainly in the issues of fatigue and sleep disorders, with 53.8% of the respondents stating 5 and above on the 7-point Likert scale for fatigue issues and with the corresponding percentage for sleep disorders being 55.6% (Table 1).

**Table 1:** Physical problems associated with parents and relatives of a child with cancer

**After the onset of my child’s illness:**				
	**I faced fatigue**		**I faced sleep disorders**	
	**Frequency**	**Percent**	**Frequency**	**Percent**
**1**	35	26.5%	26	19.5%
**2**	5	3.8%	11	8.3%
**3**	13	9.8%	7	5.3%
**4**	8	6.1%	15	11.3%
**5**	23	17.4%	23	17.3%
**6**	20	15.2%	20	15.0%
**7**	28	21.2%	31	23.3%

Regarding psychological and emotional issues, 51.1% of the respondents regarding anxiety scored the highest number 7 on the 7-point answer scale, a percentage to which if the answers of the two immediately following levels are added, it reaches 78.1% (Table 2). The feelings of fear also fluctuate at the same levels, with 58.6% at the highest level of response and a total of 82.7% stating that they experienced fear during their child’s illness (Table 2). At medium levels of 35–50% is the expression of emotions such as those of despair and the psychological fluctuations of the parents’ mood during everyday life ranges (Table 3).

**Table 2:** Psychological problems associated with parents and relatives of a child with cancer

**After the onset of my child’s illness:**				
	**I felt stress**		**I felt fear**	
	**Frequency**	**Percent**	**Frequency**	**Percent**
**1**	9	6.8%	9	6.8%
**2**	2	1.5%	5	3.8%
**3**	6	4.5%	3	2.3%
**4**	12	9.0%	6	4.5%
**5**	10	7.5%	11	8.3%
**6**	26	19.5%	21	15.8%
**7**	68	51.1%	78	58.6%

**Table 3:** Psychological and social problems associated with parents and relatives of a child with cancer

**After the onset of my child’s illness:**				
	**I felt desperation**		**I felt significant fluctuations in my mood**	
	**Frequency**	**Percent**	**Frequency**	**Percent**
**1**	40	30.1%	20	15.0%
**2**	8	6.0%	8	6.0%
**3**	15	11.3%	15	11.3%
**4**	6	4.5%	25	18.8%
**5**	16	12.0%	12	9.0%
**6**	15	11.3%	24	18.0%
**7**	33	24.8%	29	21.8%

The results regarding the personal relationship of the parents show that not only did not experience difficulty with their partners, but 61.6%, cumulatively in the three highest response, on the 7-point Likert scale, stated that they bonded significantly more after the onset of the child’s illness (Table 4). Important data are extracted from the study of managing the new reality after the onset of childhood cancer in the family. It is observed that 46.6%, cumulative to the three highest answers on the 7-point Likert scale, declare difficulty in continuing the usual daily activities (Table 5). Childhood illness also has significant implications for practical matters, such as finances. A total of 39.4% of the participants in the question of whether they faced additional expenses during their child’s illness scored the highest on the 7-point scale, to which if the next two levels are added, the cumulative percentage is 73.5% (Table 5).

**Table 4:** Intra-family and marital problems associated with parents and relatives of a child with cancer

**After the onset of my child’s illness:**				
	**My relationship with my partner was disturbed**		**My partner and I became closer**	
	**Frequency**	**Percent**	**Frequency**	**Percent**
**1**	69	51.9%	25	18.8%
**2**	19	14.3%	5	3.8%
**3**	5	3.8%	9	6.8%
**4**	14	10.5%	12	9.0%
**5**	9	6.8%	18	13.5%
**6**	7	5.3%	20	15.0%
**7**	10	7.5%	44	33.1%

**Table 5:** Practical and financial issues associated with parents and relatives of a child with cancer

**After the onset of my child’s illness:**				
	**I faced difficulty in carrying out usual activities**		**I faced additional costs**	
	**Frequency**	**Percent**	**Frequency**	**Percent**
**1**	32	24.1%	6	4.5%
**2**	15	11.3%	4	3.0%
**3**	9	6.8%	8	6.1%
**4**	15	11.3%	17	12.9%
**5**	14	10.5%	17	12.9%
**6**	16	12.0%	28	21.2%
**7**	32	24.1%	52	39.4%

## Discussion

Childhood illness, especially childhood cancer, has significant secondary effects on the parents who care for the child, including some organic disturbances in the physiology of the parents’ body. Prolonged fatigue, lack of appetite, sleep difficulties, and physical pain are symptoms that are strongly observed in parents during the period of their child’s illness [11]. The results of the study confirm similar scientific research on the effect of childhood illness on the psychosocial health of the parent [12, 13]. In a related survey, the percentages fluctuate around the same levels, with 68% stating fatigue and 51% sleep difficulties [14].

In a study focused on the impact and effects of illness within family members, where 158 studies were selected from a search of 1517 relevant results of article abstracts, it was confirmed that the family suffers significantly from the emotional burden of living with and taking care of a relative with an illness [15]. The psychological distress felt by family members often arises from feelings of helplessness and lack of control over their lives, with members expressing many and varied emotions. Guilt, anger, worry, confusion, frustration, embarrassment, despair, and loss, each of which affects each person in a different way and to a different extent, depending on the severity of the patient’s illness and the length of time that has elapsed since the day of initial diagnosis [16, 17].

In the context of intra-family changes, the relationship of the two parents is also included. In the case that a child is ill, the limited time that the couple has to devote to their personal relationship causes friction and can lead to the collapse of the relationship or the search for sexual contacts outside of marriage [18]. Of course, there are also several cases in which the couple came closer and came out stronger.

Serious effects are also noted at the socio-economic level, which can be either difficulty in attending classes or a permanent interruption of school for the siblings of children who are ill or loss of work for their parents, due to the burden of care they have to provide for them [19]. In a related study, in 8 out of 38 families with a disabled child under study, one or both parents had permanently given up their education/training or work to care for their child [20]. Additionally, the financial issues that arise when caring for patients with chronic conditions cause significant stress and mental strain [21]. Costs for treatments, transportation to doctor’s appointments, renting houses or paying for hotels in cases where the health facility is located in a different place from the permanent residence, and paying health care workers are just some examples of the multitude of expenses. It is also worth mentioning the effects on issues such as free time, personal activities, and interpersonal and friendly relationships. The combination of many obligations, stress and depression, limited financial possibilities, and the general negative mood that prevails in the family are only a few of the obstacles that its members must overcome in order to be able to find the mood to make use of the limited free time.

Regarding psychological and emotional issues, it is scientifically known that parents suffer from high levels of anxiety, fear, worry, and intense emotional strain. The numbers come to confirm previous studies [15, 17, 22, 23], something which is extremely important as the research data on these issues in our country is relatively limited. Specifically, the cumulative percentage of 78.1%, stating 5 and above on the 7-point Likert scale about anxiety in absolute number of people is the number of 104 of the 133 participants. The total percentage of 82.7%, stating 5 and above on the 7-point Likert scale about fear, translates into 110 people stating that they experienced fear during their child’s illness.

According to the expression of emotions such as those of despair and the psychological fluctuations of the parents’ mood during everyday life ranges, it is worth commenting that the picture given by the study agrees with existing other studies, in which the feeling of anxiety dominates with equal percentages of 78.1%, such as for example the relevant study by A. Lewandowska, in which the corresponding percentage is 75% [14].

Based on the literature, the appearance of childhood illness causes friction in the family and the couple and can even lead to the dissolution of the marriage, while in others, there is remarkable mutual support and cooperation between the parents, in order to face their child’s problem together, which strengthens the relationship between them [24, 25]. For example, a study showed that the father’s involvement in caring for a child with a chronic disease improves both the couple’s relationship and all intra-family relationships [26]. Such cases are often found in childhood cancer and childhood disability, where parents work together and help and support each other in order to maintain a healthy environment of family love for their sick child [27]. In the present study, the results agree with the part that supports that the parents bonded significantly more after the onset of the child’s illness, supporting each other. The same results we can find in a similar study prove that although the relationship may be more fragile after illness, increased mutual commitment was observed in some couples [28].

The results of the difficulties of managing the new reality, continuing the usual daily activities, and facing additional expenses after the onset of childhood cancer in the family agree with related studies. In one of them, mothers who cared for their children with disabilities stated that their lives were totally different from that of their friends and that they felt that the only conversations they could participate in were those about depression, thus losing a significant part of friends them and their social circle [29]. Related research has shown that the risk of psychiatric problems in families with a child with a chronic disease is three to four times greater than in families without a sick child, further emphasizing the importance of adapting to the disease, the socio-economic level of the family, the family’s social support and family functioning, as critical issues that need the attention of the therapeutic approach [30]. We have also to mention that in addition to the parents, emotionally charged with subsequent psychological, social, and impact on the level of self-reliance and functional life, are also the siblings of the sick children. The study highlighted problems of adaptation to the new reality, emotional changes, and reduction of quality of life [31]. All these studies come to help healthcare professionals understand families when they have a child with cancer and design individualized care programs to achieve optimal treatment outcomes [32].

## Conclusion

The above results testify that the immediate physical and functional problems of the child, with the repeated medical visits, the complex examinations, and the frequent hospitalizations, as well as the uncertainty about the future with the complex secondary psychological and social problems, cause stress to the child and his family, with the responsibility of treating the disease being shared between doctor, child, and family. The secondary effects of childhood illness, especially if it is a serious one such as neoplasms, constitute a threatening factor for a multitude of negative changes in the daily life of his family.
